# 非小细胞肺癌的驱动基因及其临床意义

**DOI:** 10.3779/j.issn.1009-3419.2014.06.08

**Published:** 2014-06-20

**Authors:** 岳泉 石, 向红 杨

**Affiliations:** 1 110001 沈阳，中国医科大学临床医学七年制 Seven-Year System, Clinical Medicine, China Medical University, Shenyang 110001, China; 2 110004 沈阳，中国医科大学附属盛京医院病理科 Department of Pathology, Shengjing Hospital, China Medical University, Shenyang 110004, China

**Keywords:** 肺肿瘤, 驱动基因, 临床意义, Lung neoplasms, Driver gene, Clinical significance

## Abstract

随着分子生物技术的发展和药物研发模式的转变，根据肺癌驱动基因突变谱指导分子靶向治疗已逐渐成为现实。明确各驱动基因在非小细胞肺癌（non-small cell lung cancer, NSCLC）中的发生率及是否存在优势人群，对临床实践具有重要指导意义。本文将对NSCLC中主要驱动基因的一般特点、人群特征及临床意义进行综述。

## Abstract (en)

With the development of molecular biology technology and the transforming patterns of drug research, guiding molecular targeted therapy according to the drive gene mutation spectrum in lung cancer has gradually become a reality. Definition of the mutation incidence and whether existing advantage population groups in non-small cell lung cancer (NSCLC) have important guiding significance in clinical practice. The purpose of this paper will draw a summary on the general characteristics, demographic features and clinical significance of driver genes in NSCLC.

肺癌是全球最常见的恶性肿瘤，死亡率居恶性肿瘤之首。《2013年中国肿瘤登记年报》也显示，肺癌是居我国发病率第一位的恶性肿瘤，且呈快速上升趋势。非小细胞肺癌（non-small cell lung cancer, NSCLC）占所有肺癌的80%-85%^[1]^。过去，化学治疗是晚期NSCLC的标准治疗方式之一，但传统细胞毒性药物的疗效已达到平台。近年来，通过开发靶向癌症特异基因突变的药物，NSCLC的诊断和治疗发生了重大的变革。对NSCLC体细胞突变进行常规基因检测并给予相应靶向药物治疗已逐渐贯彻到医疗实践中，给患者的长期生存带来了希望。现将NSCLC中主要驱动基因的一般特点及其人群特征综述如下。

## 1 *EGFR*基因突变

表皮生长因子受体（epidermal growth factor receptor, EGFR）是一种受体型酪氨酸激酶，是人表皮生长因子受体（human epidermal growth factor receptor, HER）家族成员之一，由胞外区（配体结合区）、跨膜区和具有酪氨酸激酶活性的胞内区组成。当表皮生长因子、转化生长因子、双调蛋白等配体与EGFR结合后，可以激活EGFR下游信号通路，从而引起肿瘤细胞的增殖、抗凋亡、侵袭和转移等。NSCLC中*EGFR*热点突变主要集中在18-21外显子，其中19外显子缺失和21外显子L858R点突变最为常见，二者都会导致酪氨酸激酶结构域活化，且都是EGFR酪氨酸激酶抑制剂（tyrosine kinase inhibitors, TKI）的敏感性突变^[2]^，外显子20的T790M突变与EGFR-TKI获得性耐药有关，还有一些突变类型的临床意义尚不明确^[3, 4]^。

目前，检测*EGFR*基因突变的方法包括：Sanger测序、焦磷酸盐测序、直接测序、蝎形探针扩增阻遏突变系统（amplified refractory mutation system, ARMS）、片段长度分析、限制片段长度多态性（restriction fragment length polymorphism, RFLP）、实时定量PCR（real-time polymerase chain reaction, RT-PCR）、高溶解曲线分析（high-resolution melting, HRM）、单碱基延伸基因分型和变性高效液相色谱（denaturing high-performance liquid chromatography, DHPLC）等，上述这些方法各有优势和劣势。《中国非小细胞肺癌患者表皮生长因子受体基因突变检测专家共识》^[5]^和《中国表皮生长因子受体基因突变和间变淋巴瘤激酶融合基因阳性非小细胞肺癌诊断治疗指南（2013版）》^[6]^推荐DNA直接测序法和ARMS法检测*EGFR*基因突变。直接测序法是直接的、可检测已知和未知突变的一种方法，过去被认为是检测基因突变的“金标准”，但其检测流程复杂漫长，污染机会多，敏感度较低（10%-30%）。ARMS法只能检测已知突变，检测流程简单快速，敏感度高，可检测样本中0.1%-1.0%的突变基因，已广泛应用于临床诊断。

NSCLC中*EGFR*基因突变率与人种相关，在北美和西欧人群中为10%左右，而在东亚人群中为30%-50%，其中在亚裔、女性、非吸烟、腺癌中*EGFR*突变率高达70%-80%^[7]^。最近一项研究^[8]^对亚裔晚期肺腺癌患者中*EGFR*基因突变情况进行了分析，结果显示，在未经选择的晚期肺腺癌患者中，*EGFR*基因突变阳性率为51.4%。根据地区进行亚组分析发现中国大陆、香港、台湾、菲律宾、越南、泰国等国家或地区*EGFR*基因突变率类似（50.2%-64.2%），而印度人群中*EGFR*突变率较低，仅为22.2%。因此，东亚人群中会有更多NSCLC患者能从EGFR-TKI治疗中获益。

EGFR-TKI是肺腺癌研究中，研究最多、证据最充分、应用最广泛的分子靶向治疗药物，已成功应用于晚期肺腺癌治疗的各个阶段。目前，我国用于NSCLC治疗的EGFR-TKI类药物主要有三种：吉非替尼（Gifitinib）、厄洛替尼（Erlotinib）和埃克替尼（Icotinib）。其中埃克替尼是我国具有完全自主知识产权的小分子靶向抗癌创新药，是继吉非替尼和厄洛替尼之后全球上市的第三个EGFR-TKI类药物^[9-11]^。由孙燕院士作为主要研究者的ICOGEN试验是埃克替尼和吉非替尼头对头的大型Ⅲ期临床试验^[12]^，是全球第一项两个EGFR-TKI之间的直接对照研究。结果显示与吉非替尼相比，埃克替尼具有相同的疗效和更少的毒副反应。埃克替尼上市为中国的适用人群创造了更多接受治疗的机会。

## 2 *KRAS*基因突变

*KRAS*属*RAS*癌基因家族成员，NSCLC中超过90%的*RAS*突变为*KRAS*突变，突变位点主要集中在12、13和61号密码子，超过90%的突变发生在12号密码子。RAS蛋白为一种GTP/GDP（三磷酸鸟苷/二磷酸鸟苷）膜结合型蛋白，*KRAS*突变可以影响GTP酶活性，从而导致RAS信号传导通路活化，引起信号传导的持续效应，使细胞具有恶性潜能最终导致细胞的恶性转化。目前，直接测序法仍是检测*KRAS*基因突变的经典方法。

*KRAS*突变约占NSCLC基因突变的15%-20%，常发生在吸烟、腺癌患者中。亚洲NSCLC患者中，*KRAS*突变频率低于北美。以往研究^[13]^证实，*KRAS*突变是肺癌的不良预后因素，*KRAS*突变患者对EGFR-TKIs治疗耐药，对常规治疗不敏感，且目前尚无上市的KRAS的靶向药物。因此，目前KRAS在NSCLC中的临床价值较为局限。但已有多项针对KRAS下游信号通路的靶向抑制剂不断进入临床试验阶段，这些试验数据将进一步揭示*KRAS*基因突变在NSCLC中的意义。

## 3 *ALK*融合基因

2007年，棘皮动物微管样蛋白4-间变淋巴瘤激酶（echinoderm microtubule associated protein like 4-anaplasitic lymphoma kinase, *EML4-ALK*）融合基因首次由日本学者发现^[14]^，这是继*EGFR*、*KRAS*基因之后在NSCLC中新发现的另一驱动基因。基因融合时，位于2号染色体上的*EML4*基因在不同位置发生断裂，调转方向，插入位于同一染色体上断裂位点相对保守的*ALK*基因的20号外显子，形成不同的融合类型，目前已发现的融合方式有10余种，其中以变异体1和变异体3最为常见^[15]^。在NSCLC中，*EML4-ALK*是*ALK*融合基因中最重要的融合形式，*ALK*还可与其他基因融合为*KIF5B-ALK*和*TGF-ALK*等，但所占比率较低。

目前检测*ALK*融合基因的方法主要有荧光原位杂交（fluorescence *in situ* hybridization, FISH）、免疫组织化学（immunohistochemistry, IHC）和实时定量PCR等^[16-18]^。FISH已被美国食品和药物管理局批准用于*ALK*融合基因检测，并作为其靶向药物克唑替尼使用的伴随诊断方法。Ventana ALK IHC（D5F3）技术平台是在常规IHC技术基础上发展起来的高敏感性检测技术，可以达到很高的准确性，该技术结果判读具有较高重复性，与FISH具有高度的检测一致性，且花费更低，已被国家食品药品监督管理总局批准用于检测*ALK*融合基因。实时定量PCR检测敏感性高，但操作步骤繁琐，且对标本的质量要求较高，但可明确具体变异体类型。以上检测方法各有优势和劣势，在实际临床应用中可能存在联用的互补性，需要根据具体情况选用合适的检测手段。

*ALK*融合基因在非选择的NSCLC患者中出现的频率较低，国外研究^[14, 19-21]^显示，在NSCLC患者中ALK融合基因阳性的发生率约为5%。国内学者^[21-23]^报道，中国NSCLC患者ALK的阳性率约为3%-11%。以往研究^[24]^表明，ALK融合基因阳性多见于年轻、不吸烟或少量吸烟、*EGFR*及*KRAS*基因突变阴性的NSCLC肺腺癌患者。在*EGFR*突变阴性的年轻、腺癌、不吸烟的肺腺癌患者中，*ALK*融合基因的表达率高达30%-40%^[25, 26]^。尽管*ALK*融合基因阳性NSCLC患者的临床病理特征与*EGFR*突变患者相似，却不能从EGFR-TKI治疗中获益。因此，在优势NSCLC人群中筛选*ALK*融合基因阳性患者并给予ALK靶向抑制剂治疗，能够大大提高诊疗效率，节约医疗成本。

克唑替尼（Crizotinib）属MET（mesenchymal-epidermal transition）、ALK和ROS1（c-ros oncogene 1）多靶点小分子酪氨酸激酶抑制剂，对*ALK*融合基因表达的肿瘤细胞具有抑制作用。研究^[27]^表明，克唑替尼能够使*ALK*融合基因阳性患者获益，明显延长患者的总生存期，2014年美国国家综合癌症网（National Comprehensive Cancer Network, NCCN）NSCLC临床指南推荐，NSCLC患者在治疗前应进行*ALK*融合基因检测，阳性患者接受克唑替尼治疗。目前，克唑替尼已在全球包括中国在内的多个国家和地区获得批准上市。2014年4月29日另一个ALK激酶抑制剂LDK378（Ceritinib）被美国食品药品管理局批准用于治疗*ALK*融合基因阳性、经克唑替尼治疗疾病进展或不能耐受的转移性NSCLC患者^[27]^。另外，一些新的ALK激酶抑制剂，如CH5424802临床试验中也表现出了较好的安全性和有效性，这些新的靶向抑制剂的研发及应用将不断推动ALK阳性NSCLC靶向治疗研究进程。

## 4 *ROS1*融合基因

人类*ROS1*基因位于6q22染色体，是胰岛素受体家族的一种跨膜酪氨酸激酶，由一个酪氨酸激酶区域、一个跨膜区域和一个含N端糖基化位点的细胞外区域组成，编码具有酪氨酸激酶活性蛋白，但其配体未知。ROS1重排位点主要发生在32-36外显子，在NSCLC中*ROS1*基因主要与SCL34A2、CD74发生融合，但也可与SDC4、TMP3、EZR等融合。目前检测*ROS1*融合基因的方法与检测*ALK*融合基因方法类似，主要包括FISH、IHC和实时定量PCR等，文献报道的各种检测方法的敏感性、特异性及一致性也存在较大差异，因此与检测*ALK*融合基因类似，在实际临床应用中各种检测方法存在联用的互补性。

作为新发现的肺癌驱动基因，明确*ROS1*融合基因在NSCLC中的发生率及是否存在优势人群，对临床实践具有重要指导意义。*ROS1*融合基因在非选择NSCLC患者中阳性比率很低，仅占1%-2%，且好发生于年轻、女性、非吸烟的腺癌患者中，与*EGFR*、*KRAS*及*ALK*融合基因互斥^[28-31]^。Mescam-Mancini等^[32]^在121例*EGFR*、*KRAS*及*ALK*三阴性法国患者中检测*ROS1*融合基因，结果发现有9例（7.4%）患者ROS1 IHC和FISH双阳性。Kim等^[33]^在162例非吸烟Ib期到IIIa期韩国患者中检测*EGFR*、*KRAS*、*ALK*及*ROS1*融合基因，*ROS1*融合基因的阳性率为3.2%，*EGFR*、*KRAS*、*ALK*三阴性患者中，*ROS1*融合基因的阳性率高达8.3%。另一项研究^[34]^对202例中国非吸烟肺腺癌患者中采用RT-PCR检测*ROS1*融合基因，阳性率为1%，*EGFR*、*KRAS*、*HER2*、*ALK*四阴性患者中*ROS1*阳性率为8.3%。因此，在这些优势人群中筛选*ROS1*融合基因阳性患者，检出率将会有所提高。但*ROS1*融合基因是否像*EGFR*基因突变一样存在种族差异，目前尚无定论。

ROS1与ALK同属胰岛素样受体酪氨酸激酶超家族成员，二者的激酶结构域间约有49%氨基酸序列同源性，激酶结构域ATP-结合位点区域的同源性高达77%^[35]^。因此，ALK激酶抑制剂也可抑制ROS1激酶活性。Shaw等^[36]^对ROS1阳性的接受克唑替尼治疗的14例可评价的患者疗效进行评价，12例患者可明显获益。提示克唑替尼对*ROS1*融合基因阳性NSCLC患者有值得期待的治疗潜力。随着新的ALK激酶抑制剂的出现，必将会有越来越多的*ROS1*融合基因阳性患者从靶向治疗中获益。

## 5 *MET*（mesenchymal-epidermal transition）基因扩增

*MET*基因位于7号染色体7q31区，编码的肝细胞生长因子（hepatocyte growth factor, HGF）特异性受体属酪氨酸激酶型受体。HGF与MET受体结合后受体激活发生自身磷酸化，引起多种底物蛋白磷酸化并引起细胞内一系列信号传导，激活PI3K/AKT及MET/ERK等信号通路，促进肿瘤细胞的生长、侵袭和转移^[37, 38]^。MET是NSCLC的不良预后因素之一且其介导的信号通路与EGFR的信号通路存在交叉，NSCLC中，MET扩增是EGFR-TKI获得性耐药的主要原因之一，约占5%-12%。因此，在NSCLC中MET扩增研究是新近的热点。

MET是克唑替尼抑制靶点之一，体外实验证实，克唑替尼在MET扩增的肺癌细胞中有较强的抗肿瘤活性，但对不扩增的肺癌细胞无效。目前，已有克唑替尼治疗MET扩增NSCLC患者有效病例。2014年美国临床肿瘤学会会议上报告了克唑替尼治疗晚期*MET*基因扩增型NSCLC的疗效和安全性数据，研究结果^[39]^显示，克唑替尼在*MET*基因扩增型NSCLC患者中呈现了抗肿瘤活性，患者普遍能够耐受，而不良事件亦在可接受范围。这些研究结果值得对克唑替尼治疗晚期MET扩增型的NSCLC作进一步研究。其他一些针对MET扩增的抑制剂如cabozantinib和foretinib等也陆续进入临床试验阶段，相信这些药物的研究进展将为MET基因扩增型NSCLC患者带来生存福音。

## 6 *FGFR1*基因扩增

成纤维细胞生长因子受体1（fibroblast growth factor receptor 1, FGFR1）属酪氨酸激酶跨膜受体，2010年首次在肺鳞癌中发现^[40]^。目前研究^[41, 42]^显示，肺鳞癌中FGFR1扩增率约为20%，在腺癌里发生比率很低。另外，FGFR1扩增与吸烟及吸烟剂量呈现一定相关性^[42]^，吸烟可能通过破坏FGFR1蛋白编码基因而导致肺鳞癌的发生。但以上研究人群主要为西方人群，中国肺鳞癌患者中*FGFR1*基因扩增频率及临床病理特点尚需进一步研究。目前尚无针对FGFR1扩增的靶向抑制剂上市，但一些针对FGFR1的靶向药物如多韦替尼、AZD4547、BGJ398等也陆续进入临床试验阶段，这些试验数据有望为肺鳞癌靶向治疗带来突破。

## 7 *DDR2*基因突变

盘状结构域受体（discoidin domin receptor tyrosine kinase 2, DDR2）属酪氨酸激酶受体，其表达上调与NSCLC，尤其是鳞癌无病生存时间和总生存时间延长相关^[43]^。DDR2在肺鳞癌中的发生率不高，接近于肺腺癌中*ALK*融合基因的发生率。但初步研究数据表明，一些DDR2抑制剂如达沙替尼对肺鳞癌*DDR2*突变患者有较好疗效，这些抑制剂的临床试验数据将为*DDR2*突变肺鳞癌患者长期生存带来希望。

## 8 结语

随着EGFR-TKI和ALK抑制剂在NSCLC患者中的成功应用，针对*EGFR*基因突变、*ALK*融合基因阳性肺癌的诊疗模式已成功建立，这也为其他驱动基因的研究进程提供了可参考范例。随着肿瘤分子标志物检测手段的不断成熟及靶向药物研发模式的转变，我们必将迎来NSCLC个体化治疗的全新突破。
